# Simultaneous mixed phenotype and neuroimaging of progressive supranuclear palsy, progressive ataxia and palatal tremor: two different faces of tauopathies

**DOI:** 10.1590/0004-282X-ANP-2021-0442

**Published:** 2022-08-08

**Authors:** Leonardo Furtado Freitas, Victor Moreira de Carvalho, José Luiz Pedroso, Márcio Luís Duarte, Rodrigo Meirelles Massaud

**Affiliations:** 1 Universidade Federal de São Paulo, Departamento de Radiologia, São Paulo SP, Brazil.; 2 Beneficência Portuguesa de São Paulo, Departamento de Radiologia, São Paulo SP, Brazil.; 3 Universidade Federal de São Paulo, Departamento de Neurologia, São Paulo SP, Brazil.; 4 Universidade Federal de São Paulo, Departamento de Saúde Baseada em Evidências, São Paulo SP, Brazil.; 5 Hospital Israelita Albert Einstein, Departamento de Neurologia, São Paulo SP, Brazil.

**Figure 1.**
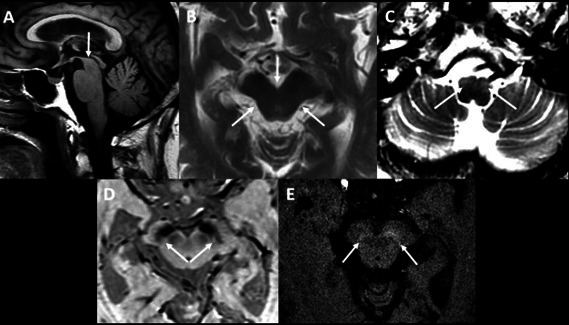
3.0 Tesla magnetic resonance imaging shows volumetric reduction of the midbrain in the sagittal T1 sequences (A), with rectification of the upper contour of the tegment (arrow) and axial T2 (B) showing prominence of the interpeduncular cistern and greater concavity of the tegment (morning glory sign - arrows) described in progressive supranuclear palsy (PSP). The T2 axial sequence of the medulla oblongata (C) shows hypersignal and hypertrophy of bilateral inferior olivary nuclei (arrows), which can be associated to progressive ataxia and palatal tremor (PAPT). Axial thin slices of the midbrain in SWI (D) and T1 (E) sequences with reduction of nigrosome1 and neuromelanin in the substantia nigra (arrows), respectively, mainly on the right, a structural biomarker of transaxonal degeneration of the striatonigral dopaminergic pathway, not specific for this entity, which can found in other parkinsonian syndromes, such as Parkinson's disease.

A 74-year-old woman was referred with a six-month history of progressive gait disturbance and falls. Physical examination showed bradykinesia, rigidity, postural instability, palatal tremor, and vertical gaze palsy (Video). There was no improvement with levodopa. Brain magnetic resonance demonstrated midbrain atrophy and hyperintense signal in the inferior olivary nuclei (Figure 1). The patient was diagnosed simultaneously with progressive supranuclear palsy (PSP), progressive ataxia and palatal tremor (PAPT).

**Video.** Patient with mixed phenotype: progressive supranuclear palsy, progressive ataxia and palatal tremor. Note gait instability, ataxia, bradykinesia, tremor, vertical gaze palsy and palatal tremor. 

PSP is a well-known form of tauopathy. Recent reports have described tau-positive neuronal inclusions in PAPT^1^. The unusual syndrome characterized simultaneously by PSP and PAPT may suggest a unique phenotype of tau pathology^2^. 
